# Vibrational Spectroscopy for the Triage of Traumatic Brain Injury Computed Tomography Priority and Hospital Admissions

**DOI:** 10.1089/neu.2021.0410

**Published:** 2022-06-03

**Authors:** Ashton G. Theakstone, Paul M. Brennan, Katherine Ashton, Endre Czeiter, Michael D. Jenkinson, Khaja Syed, Matthew J. Reed, Matthew J. Baker

**Affiliations:** ^1^ Department of Pure and Applied Chemistry, University of Strathclyde, Glasgow, United Kingdom.; ^2^ Translational Neurosurgery, Centre for Clinical Brain Sciences, University of Edinburgh, Edinburgh, United Kingdom.; ^3^ Neuropathology, Lancashire Teaching Hospitals NHS Trust, Royal Preston Hospital, Preston, United Kingdom.; ^4^ Department of Neurosurgery, Medical School and Szentágothai Research Centre, University of Pecs, Pecs, Hungary.; ^5^ Neurotrauma Research Group, Szentágothai Research Centre, University of Pecs, Pecs, Hungary.; ^6^ MTA-PTE Clinical Neuroscience MR Research Group, Pecs, Hungary.; ^7^ The Walton Centre NHS Foundation Trust, Liverpool, United Kingdom.; ^8^ Department of Pharmacology & Therapeutics, Institute of System, Molecular and Integrative Biology, University of Liverpool, Liverpool, United Kingdom.; ^9^ Emergency Medicine Research Group Edinburgh (EMERGE), Department of Emergency Medicine, Royal Infirmary of Edinburgh, Edinburgh, United Kingdom.; ^10^ Dxcover Ltd, Glasgow, United Kingdom.

**Keywords:** chemometrics, CT brain imaging, traumatic brain injury, vibrational spectroscopy

## Abstract

Computed tomography (CT) brain imaging is routinely used to support clinical decision-making in patients with traumatic brain injury (TBI). Only 7% of scans, however, demonstrate evidence of TBI. The other 93% of scans contribute a significant cost to the healthcare system and a radiation risk to patients. There may be better strategies to identify which patients, particularly those with mild TBI, are at risk of deterioration and require hospital admission. We introduce a blood serum liquid biopsy that utilizes attenuated total reflectance (ATR)-Fourier transform infrared (FTIR) spectroscopy with machine learning algorithms as a decision-making tool to identify which patients with mild TBI will most likely present with a positive CT scan. Serum samples were obtained from patients (*n* = 298) patients who had acquired a TBI and were enrolled in CENTER-TBI and from asymptomatic control patients (*n* = 87). Injury patients (all severities) were stratified against non-injury controls. The cohort with mild TBI was further examined by stratifying those who had at least one CT abnormality against those who had no CT abnormalities. The test performed exceptionally well in classifications of patients with mild injury versus non-injury controls (sensitivity = 96.4% and specificity = 98.0%) and also provided a sensitivity of 80.2% when stratifying mild patients with at least one CT abnormality against those without. The results provided illustrate the test ability to identify four of every five CT abnormalities and show great promise to be introduced as a triage tool for CT priority in patients with mild TBI.

## Introduction

Population-based studies from numerous countries have estimated the global incidence of traumatic brain injury (TBI) to be 50–60 million new cases annually. In the United Kingdom (UK), in England and Wales ∼1.4 million persons with TBI visit the Emergency Department (ED) annually.^1^ Of these, the majority (∼68%) have a mild TBI,^2^ as defined as a score of 13 to 15 on the Glasgow Coma Scale (GCS).^2,3^

Optimal management benefits from accurate assessment of TBI severity and prediction of likely outcome, most commonly from assessment of consciousness. Consciousness is most often assessed with the GCS. Combination of GCS with other variables—for example, pupil responses (GCS-P)—can enhance prediction of six-month death and functional impairment.^4^ A combination of GCS-P, patient age, and CT findings further benefits outcome predictions, using a combined effects approach.^5^ These outcome prediction tools are most useful in the moderately and severely brain injured patient. In mild head injury, poor outcomes are very infrequent, and outcomes of interest are more likely to be hospital admission or length of stay.

Computed tomography (CT) brain scanning is a standard investigation to identify intracranial pathology that might portend clinical deterioration and possible requirement for operative intervention, or to exclude significant pathology and expedite discharge. The CT imaging followed by at home self-observation can reduce healthcare costs by avoiding unnecessary hospital admission of those with mild TBI. As reported by Geijerstam and associates,^6^ the option of CT followed by earlier discharge can save up to £280,000 per million in the UK's National Health Service (NHS) as opposed to in-hospital observations.

An estimated 7% of patients who undergo CT imaging have evidence of TBI^7^ and therefore a large proportion of the remaining 93% of scans may be unnecessary. Further, all patients are exposed to radiation, which confers a brain tumor risk; even as few as 2–3 scans as a child can triple the risk, and each additional CT scan increases the incidence rate ratio by 0.16.^8^

Strategies to triage patients with mild head injury are needed that reduce radiation exposure and unnecessary imaging while maintaining economic savings from early discharge. This is especially important given the increasingly lower thresholds for CT, especially in patients receiving antiplatelet medication, advocated in recent head injury guidelines.^1^ Predictors of high risk in mild head injury include headache, loss of consciousness/amnesia, and alcohol intoxication,^9^ but lack both sensitivity and specificity.

Numerous blood-based biomarkers have been assessed as prognostic markers, but with little clinical impact. In 2018 the U.S. Food and Drug Administration (FDA) approved the use of glial fibrillary acidic protein (GFAP) and ubiquitin carboxy-terminal hydrolase L1 (UCH-L1) assays to aid in TBI evaluations.^10,11^ Handheld devices such as the Abbott i-STAT^TM^ have been developed for rapid, point-of-care measurements; however, prognostic impact currently remains largely unknown.^11^

The ALERT-TBI study assessed these two blood-based brain biomarkers (GFAP and UCH-L1) in 1977 in patients presenting a GCS score of 9–15 within 12 h of injury.^12^ A combination of the two biomarkers predicted CT abnormalities with high sensitivity, but low specificity.^12^ More recently, a study from the Collaborative European NeuroTrauma Effectiveness Research in TBI (CENTER-TBI) team explored the relationship between six different blood biomarkers (S100B, neuron-specific enolase [NSE], GFAP, UCH-L1, neurofilament light chain protein [NfL], and t-tau) with brain injury severity and CT findings.^13^ Only GFAP performed well in predicting CT abnormalities (area under the curve [AUC] = 0.89), but requires external validation.^13^

There is a need for a rapid, low cost, sensitive and specific test to triage head injured patients in the ED. Rather than assaying single molecules, our strategy uses attenuated total reflectance-Fourier transform infrared (ATR-FTIR) spectroscopy for assessment of more than 20,000 molecules in serum, surveying the brain and systemic response to head injury. This phenotypic method quantifies molecular absorption of mid-infrared light and results in a spectrum that provides information on the overall composition of the sample.^14^ Molecules such as lipids, carbohydrates, nucleic acids, and proteins are easily recognizable by absorption at specific wavenumber regions.^14–16^

The methodology is rapid, commercially available, and involves silicon internal reflection elements (SIREs), which allows for multiple sampling points, is relatively low cost, and suitable for investigative use at high throughput.^17–19^ Our group previously demonstrated this platform technology performs with high sensitivity and specificity for brain tumor detection in symptomatic patients.^20^ We therefore hypothesized that spectral data from patient serum can be used as a triage method to identify patients with mild TBI most likely to have a CT abnormality.

## Methods

Two hundred and ninety-eight serum samples were obtained from CENTER-TBI for spectroscopic analysis. The CENTER-TBI study was a prospective observational clinical study conducted in 65 sites from 17 European countries and Israel between December 19, 2014, and December 17, 2017. Patients with all severities of TBI presenting to a study center within 24 h of injury and scheduled for CT scanning were enrolled. The only exclusion criterion was severe pre-existing neurological disorder.

All the measured 298 samples were randomly selected on admission samples from the CENTER-TBI serum biobank, collected within 24 h post-injury in 11 of the 65 participating clinical sites of the CENTER-TBI project. The samples underwent two freeze-thaw cycles before analysis, because serum aliquots were primarily used for other biomarker tests by the CENTER-TBI group. Of these, 222 patients were analyzed who had both GCS scores recorded from the time of injury and Extended Glasgow Outcome Scale (GOSE) values at six months post-injury. The 222 cohort samples were compared with those of 87 asymptomatic controls whose serum samples were obtained from Royal Preston Hospital (Preston, UK).

The TBI patient cohort had a mean age of 50.7 years and were represented in a 70:30% male to female ratio. The asymptomatic controls had a mean age of 34.4 years and a 55:45% female to male ratio. At the time of injury, 49% (108) of patients presented with a mild head injury, 14% with moderate head injury, and 37% with severe head injury. These classifications were based solely on GCS score: mild 13–15, moderate 9–12, and severe 3–8.

From the CT imaging, 23% of patients with TBI had no abnormalities. The remaining 77% with abnormalities presented with one or more of the following six: small hyperdense lesions, extradural hematoma, acute subdural hematoma, contusion, subarachnoid hemorrhage, or basal cisterns absent compressed. Six months post-injury, 21% of patients had died as a result of their injury, while 20%, 18%, and 41% had severe, moderate, or good recoveries, respectively. A summary of patients included within the study is outlined in Table 1.

**Table 1.** Patients Included within the Study

Factor	Value (%)
No. of injury patients	222
Age range (years)	3–92
Mean age (years)	50.7
IQR (years)	34
Gender M/F	156/66 (70/30)
GCS score	
13–15 (Mild)	108 (49)
9–12 (Moderate)	32 (14)
3–8 (Severe)	82 (37)
6-month GOSE	
Mortality	46 (21)
Severe disability	44 (20)
Moderate disability	41 (18)
Good recovery	91 (41)
No. of healthy patients	87
Age range (years)	20 - 69
Mean age (years)	34.4
IQR (years)	17
Gender M/F	39/48 (45/55)

IQR, interquartile range; GCS, Glasgow Coma Scale; GOSE, extended Glasgow Outcome Scale.

A Perkin Elmer Spectrum 2 FTIR spectrometer (Perkin Elmer, UK) was used for all serum spectral data collection. This involved a Specac Quest ATR accessory unit with a specular reflectance puck (Specac Ltd, UK), allowing a Dxcover optical sample SIRE (Dxcover Ltd) to be positioned directly on top of the aperture. Nine spectra were collected for each patient within the wavenumber range of 4000–450 cm^−1^, at a resolution of 4 cm^−1^, with 1 cm^−1^ data spacing and 16 co-added scans; resulting in a total of 2781 spectra acquired. For nine repeats and background, each patient took approximately 15 min for data collection.

The data analysis was completed using either MATLAB R2020a or R Statistical Computing Environment software with the PRFFECT toolbox,^21^ a principal component analysis (PCA) code written in house, a receiver operating characteristic (ROC) curve code written in house, or a partial least squares-discriminant analysis (PLS-DA) bootstrapping code for permutation analysis. 

Data pre-processing was a trial-and-error iterative approach utilizing the PRFFECT toolbox and was completed before each PCA or classification. The optimum pre-processing techniques for these data involved a min max normalization, a binning factor of 8, cutting to the spectral region of 1800–1000 cm^−1^, and an extended multiplicative signal correction (EMSC) that used the average spectrum of 10 background measurements of the SIRE as a reference.

Three classifications within the PRFFECT toolbox were chosen including random forest (RF), PLS-DA, and support vector machine (SVM). Patients were split randomly into training (70%) and test sets (30%) where the models were tuned on the training set and then used to make predictions for the spectra within the test set. A five-fold cross-validation on the training set was performed on a per-spectra basis. To ensure that the models were trained and validated correctly, spectra from a single patient's sample could only appear in one cross-validation fold, and in either the training or test set. The consensus vote among the nine spectra that were analyzed for each patient was reported as the diagnostic outcome. The 51 reiterations reshuffle the training and test sets to ensure all patients are included within the test set at least once.

Because of the imbalance between the two groups (injury and non-injury), a synthetic minority oversampling technique (SMOTE) was used for all classifications. The SMOTE sampling technique is an oversampling approach that creates synthetic data for the minority class to create a class balance, and it is used widely for high dimensional data.^22^ Sensitivity, specificity, and balanced accuracies contribute to the performance of each algorithm^23^ while a ROC curve can measure performance capabilities and the AUC represents a degree of separability.^24^ The statistical significance of the classification was obtained by an empirical *p* value, where 1000 permutation tests with randomized labels were completed. This was done with a PLS-DA classification model with SMOTE sampling and 1000 bootstrapping validations.^25^

Analysis first involved comparison between injury patients (all severities) and a healthy volunteer non-injury control group. Following this, patients who presented with a mild GCS score were compared with non-injury controls, and, finally, comparisons were made between patients with mild GCS scores with at least one CT abnormality against mild patients with GCS scores with no CT abnormalities. Patient age and gender were also investigated to establish any influence on test accuracy because of these two variables.

## Results

**FIG. 1.**
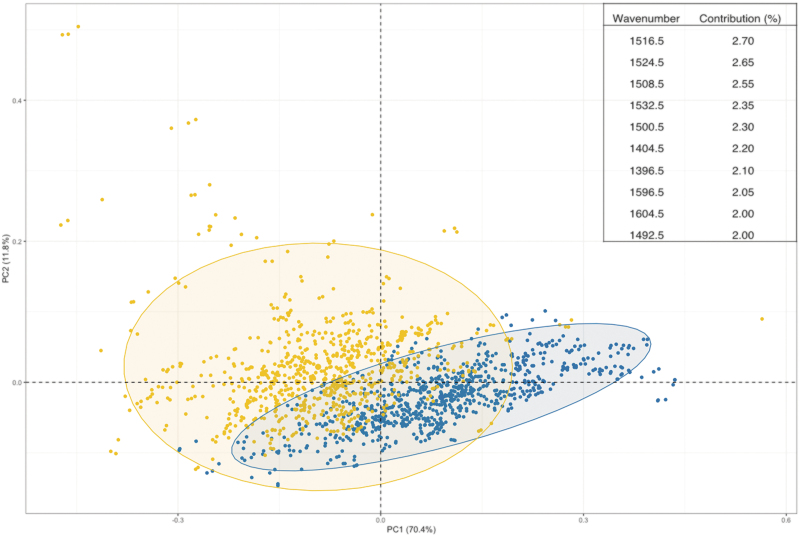
Principal component analysis (PCA) of the first and second dimensions with all injury patients in yellow and healthy controls in blue. The eclipses represent a 95% confidence interval. Values in parentheses are the total explained variance in each PC. Inset are the top 10 wavenumbers that contribute to the separation of the two classes within the first dimension. Color image is available online.

Exploratory and classification analysis was completed on the total cohort (*n* = 222) of head injury patients (all severities) against non-injury controls (*n* = 87). For the exploratory analysis, PCA was completed to explain any variance between the two classes of patients (head injury and non-injury) and is shown in Figure 1. The PCA plot illustrates a separation between the injury and non-injury patients along the first PC with the top 10 wavenumbers responsible for this separation highlighted. The top 10 wavenumbers range between approximately 1400 cm^−1^ and 1600 cm^−1^, which demonstrates that the variation between classes comes from the Amide I and Amide II region of the spectrum.

Three different classification models (PLS-DA, SVM, and RF) were explored to stratify between the two classes, with PLS-DA resulting in the greatest predictive ability. Sensitivity (96.0%), specificity (98.1%), and balanced accuracy (97.1%), shown in Table 2, are all greater than 95% in stratifying between injured and non-injured patients.

**Table 2.** Sensitivity, Specificity, and Balanced Accuracies for All Patients with Head Injury versus Controls with the Partial Least Squares-Discriminant Analysis, Random Forest, and Support Vector Machine Classification Models with 95% Confidence Intervals Included

Model	Sensitivity (%)			Specificity (%)			Balanced accuracy (%)		
	Mean	SD	95% CI	Mean	SD	95% CI	Mean	SD	95% CI
PLS-DA	96.0	3.4	±0.9 95.1–96.9	98.1	2.6	±0.7 97.4–98.8	97.1	2.1	±0.6 96.5–97.7
RF	95.7	3.9	±1.1 94.6–96.8	95.3	4.0	±1.1 94.2–96.4	95.6	2.5	±0.7 94.9–96.3
SVM	97.5	2.9	±0.8 96.7–98.3	95.7	4.2	±1.2 94.5–96.9	96.6	2.5	±0.7 05.9–97.3

SD, standard deviation; CI, confidence interval; PLS-DA, partial least squares-discriminant analysis; RF, random forest; SVM, support vector machine.

**FIG. 2.**
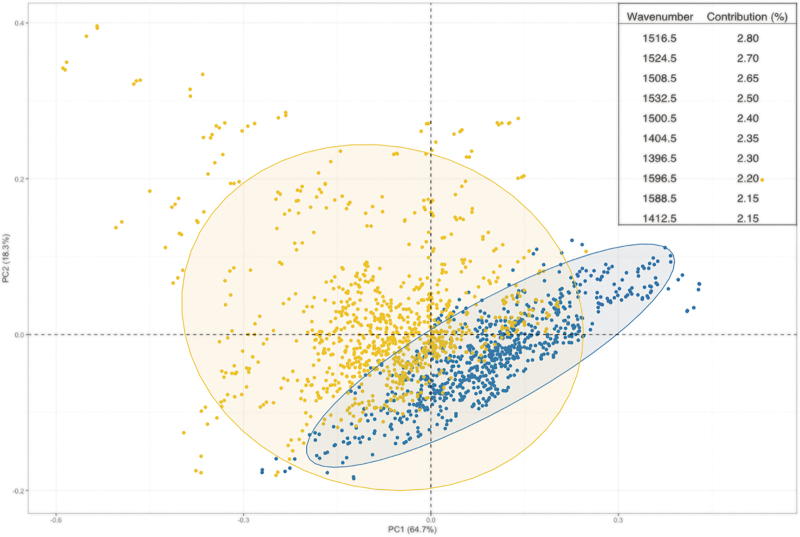
Principal component analysis (PCA) of the first and second dimensions with patients with mild injury in yellow and healthy controls in blue. The eclipses represent a 95% confidence interval. Values in parentheses are the total explained variance in each PC. Inset are the top 10 wavenumbers that contribute to the separation of the two classes within the first dimension. Color image is available online.

We next examined patients with mild head injury symptoms. Exploratory and classification analysis was completed on a group of patients (*n* = 108) who were categorized as mild through their GCS rating. The PCA plot of mild injuries against non-injuries illustrated a similar trend to the total cohort, with a slight separation between the classes within the first dimension. The wavenumbers that are deemed important for this separation correspond well with the total cohort, reiterating the importance of the Amide I and Amide II spectral region for discrimination (Fig. 2).

THE PLS-DA once again performed the greatest of the three classification models (PLS-DA, SVM, and RF) with sensitivity (96.4%), specificity (98.0%), and balanced accuracy (97.2%) remaining above 95%, as shown in Table 3.

**Table 3.** Sensitivity, Specificity, and Balanced Accuracies for Patients with Mild Head Injury versus Controls with the Partial Least Squares-Discriminant Analysis, Random Forest, and Support Vector Machine Classification Models with 95% Confidence Intervals Included

Model	Sensitivity (%)			Specificity (%)			Balanced accuracy (%)		
	Mean	SD	95% CI	Mean	SD	95% CI	Mean	SD	95% CI
PLS-DA	96.4	3.7	±1.0 96.4–97.4	98.0	2.9	±0.8 97.2–98.8	97.2	2.2	±0.6 96.6–97.8
RF	95.5	3.3	±0.9 94.6–96.4	97.4	2.7	±0.7 96.7–98.1	96.4	2.2	±0.6 95.8–97.0
SVM	96.3	3.5	±1.0 95.3-97.3	93.8	4.0	±1.1 92.7–94.9	95.0	2.7	±0.7 94.3–95.7

SD, standard deviation; CI, confidence interval; PLS-DA, partial least squares-discriminant analysis; RF, random forest; SVM, support vector machine.

**FIG. 3.**
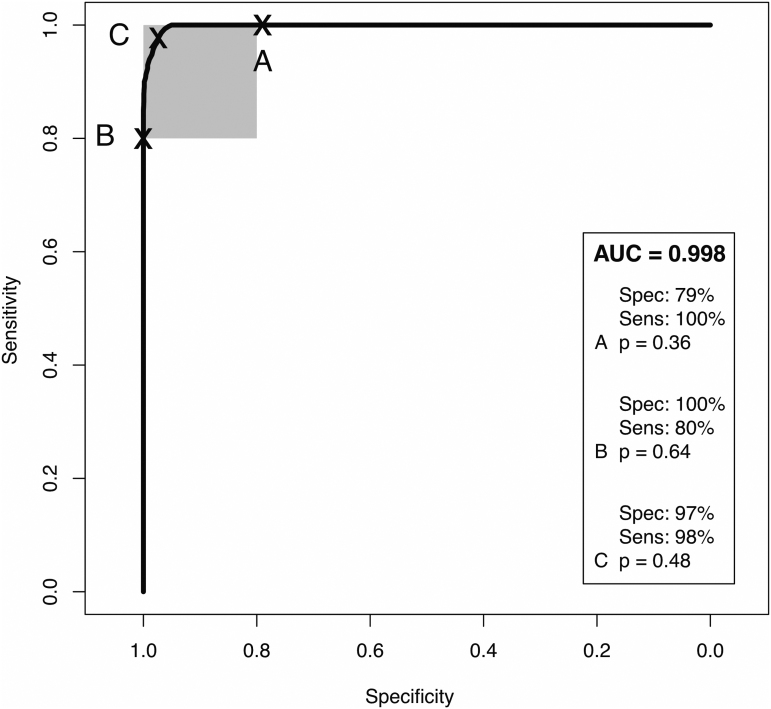
Receiver operating characteristic curves with area under the curve (AUC) for patients with mild head injury classified against healthy controls. Nine patient repeats and 51 reiterations with partial least squares-discriminant analysis model.

For the mild head injury patients and non-injury controls, a ROC curve was completed to display the diagnostic capability of the PLS-DA classification model, with the AUC calculated as 0.998 (Fig. 3). The classification model can be tuned by altering the threshold (p) to favor either sensitivity or specificity. Point A represents the greatest sensitivity, point B represents the greatest specificity, and point C is the optimum threshold for both excellent sensitivity and specificity.

**FIG. 4.**
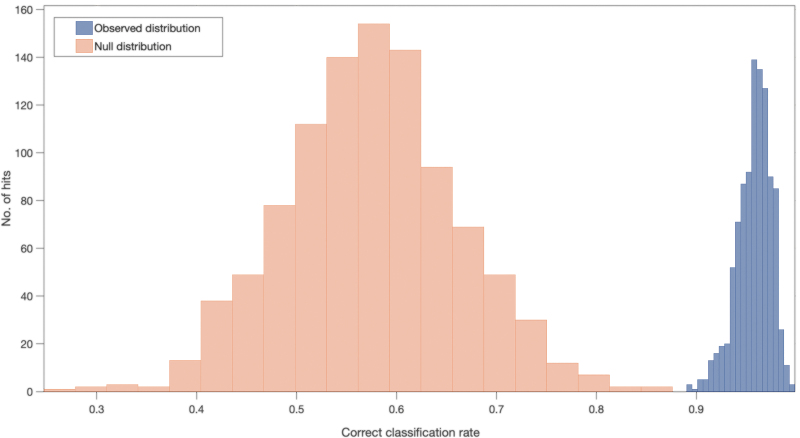
Null (orange) and observed (blue) distribution classification rates for patients with mild head injury against healthy controls with a partial least squares-discriminant analysis classification model after 1000 bootstraps. Color image is available online.

A PLS-DA classification model with 1000 bootstrapping validations was completed to assess the statistical significance of the classification findings and to complete a permutation analysis. The correct classification rate (CCR) for the patients with mild injury versus controls was calculated as 0.96, which demonstrates excellent separation between the null and observed distributions. Figure 4 illustrates the null and observed distributions where a clear separation is visible, supporting the machine learning classifications. The null hypothesis (which states that the separation happened by chance) can be rejected because the classification results are deemed genuine. A *p*-value was calculated (*p* = <0.001) from this analysis to demonstrate statistical significance and to give further evidence against the null hypothesis.

**FIG. 5.**
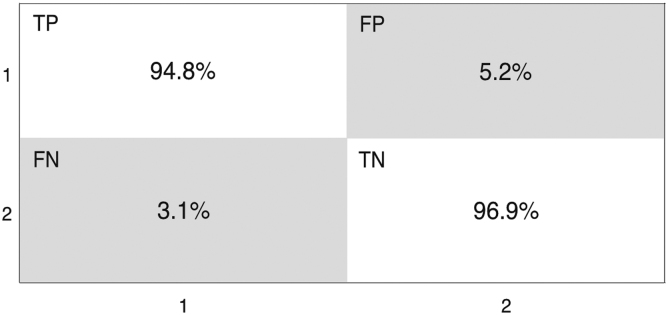
Confusion matrix illustrating the percentage of true positive (TP), false positive (FP), false negative (FN), and true negative (TN) between the patients with mild head injury (Class 1) and healthy controls (Class 2). Partial least squares-discriminant analysis classification model after 1000 bootstraps.

A confusion matrix calculates the true positives (TP), false positives (FP), true negatives (TN), and false negatives (FN) of the two classes from the machine learning classification. Class 1 represents the mild injury patient cohort while class 2 is the non-injury controls. From the confusion matrix (Fig. 5), the TP represents 94.8% of patients with mild injury correctly identified, with 5.2% assumed to be a non-injury control and identified as a FP. The TN represents 96.9% of non-injury controls correctly identified with 3.1% deemed to have a mild injury and is a FN.

The mild injury cohort was further explored by separating the group into two subgroups: patients who presented with no CT abnormalities and patients with at least one CT abnormality. These two subgroups were subjected to the same exploratory and classification analysis. The unsupervised PCA indicated little separation between the two (Supplementary Fig. S1), while the classification model PLS-DA gave high sensitivity and lower specificity as outlined in Table 4.

**Table 4.** Sensitivity, Specificity, and Balanced Accuracies for Patients with Mild Head Injury with at Least One Computer Tomography Abnormality versus Patients with Mild Head Injury with No Computer Tomography Abnormalities. Partial Least Squares-Discriminant Analysis, Random Forest, and Support Vector Machine Classification Models with 95% Confidence Intervals Included

Model	Sensitivity (%)			Specificity (%)			Balanced accuracy (%)		
	Mean	SD	95% CI	Mean	SD	95% CI	Mean	SD	95% CI
PLS-DA	80.2	9.2	±2.5 77.7–82.7	33.2	14.4	±4.0 29.2–37.2	56.7	7.2	±2.0 54.7–58.7
RF	62.5	14.1	±3.9 58.6–66.4	46.6	15.3	±4.2 42.4–50.8	54.5	8.5	±2.3 52.2–56.8
SVM	61.1	13.4	±3.7 57.4–64.8	42.8	17.9	±4.9 37.9–47.7	52.0	10.0	±2.7 49.3–54.7

SD, standard deviation; CI, confidence interval; PLS-DA, partial least squares-discriminant analysis; RF, random forest; SVM, support vector machine.

The high sensitivity illustrates that this technique correctly identifies CT abnormalities in 80% of the patients with mild head injury who do, in fact, have a CT abnormality. The lower specificity indicates that only one third of patients indicated by the blood test to have a CT abnormality will actually have one on brain imaging.

Finally, an exploratory and classification analysis was completed on two patient variables (gender and age) to prove their hypothesized lack of influence on these classification models to ensure the methods developed here are applicable to all patients with head injury. Exploratory PCA as well as the three classification algorithms (PLS-DA, SVM, and RF) were completed when patients were separated by gender or age. The PCA plot of female and male patients (Supplementary Fig. S2) shows no separation between the two classes. A similar result is observed with patients under 40 years of age were compared with patients more than 60 years of age (Supplementary Fig. S3).

The classification results have sensitivities, specificities, and balanced accuracies less than 70% (Supplementary Table S1). This indicates little variations between the classes and can confidently suggest that patient age or gender does not influence the classification of mild injuries and will not need to be considered an influencing variable with patient assessment.

## Discussion

We have demonstrated that we can achieve accurate discrimination of patients with head injury from non-injury controls using ATR-FTIR. This technique proved exceptional for the stratification of all severities versus controls as well as mild only versus controls. Screening the entire serum composition clearly identifies the differences that are likely because of the body's systemic response to the injury acquired. Stratifying mild patients further into CT positive or CT negative proved less accurate, however; the high sensitivity accounts for 80% of all CT abnormalities being identifiable with this method. The optimal balance of sensitivity and specificity from model calibration may vary depending on local triage pathways and clinical preferences for reducing FN and FP results.

Because 93% of brain images performed in TBI are predicted to be “normal,” this technique has the potential to reduce hospital admissions, CT scans, and costs, and limit exposure to ionizing radiation. Future studies will explore enhancing test accuracy through the combination of patient and clinical features with spectroscopy data, as well as refinement of the machine learning algorithm.

While 7% of CT scans demonstrate evidence of TBI, there is an array of CT findings in TBI including hematoma collection and diffuse contusional injury. Many patients with mild CT TBI changes will not require advanced head injury care and/or operative management and will make an uneventful recovery with little intervention. Similarly, there are some patients without CT TBI changes that have persistent post-head injury concussional symptoms who have changes on other imaging modalities such as MRI. It is as yet unknown whether our test is able to better identify patients with more significant CT abnormalities and also whether the FP signals identified by the blood test may, in fact, indicate brain injury unable to be identified on CT.

While the clinical value of our test is likely to be in better acute triage of TBI and possible rationalization of CT brain imaging, it remains to be seen whether it may also have a role in prognostication and prediction of patients at risk of longer term complications. A potential limitation of our study is that we considered the significance of CT brain imaging based simply on the number of abnormalities. This risks misclassifying the severity of any individual abnormality. We previously demonstrated, though, in a study of more than 10,000 patients with TBI, that in a trade-off between predictive yield and simplicity, reducing the number of CT groupings maintained most of the predictive yield.^5^

Current clinical assessment of TBI is heavily dependent on CT brain imaging, despite many scans being unremarkable and few identified abnormalities impacting patient care. Patients might be better assessed without the risks and cost of ionizing radiation.^26^ The potential risks of CT radiation exposure have been correlated with cancer incidence. Pearce and coworkers^8^ retrospectively investigated patients in the UK who had CT brain imaging between 1985 and 2002. They identified a significant linear association with imaging and brain tumors.^27^ Reducing CT imaging reduces this risk.^28,29^

Using patient serum, this technique provides a rapid, low-cost stratification of the likelihood of CT abnormality. In patients with mild head injury, this permits assessment before brain injury referral. Importantly, factors such as patient age and gender do not influence test results and therefore can be widely applied to all patients with TBI. The healthy volunteer control group in our study was not age and gender matched to the TBI group. This will not have impacted the study outcome.

In future prospective validation studies, we will explore performance of the test algorithm in patients with TBI compared with non-TBI patients with other cranial pathology, such as stroke and brain tumor. While blood biomarker tests do not replace thorough clinical assessment and decision making, the test is an adjunct to this process. Future work will assess the effect on test performance in combination with current assessment techniques, such as GCS and pupil reactivity.

## Conclusion

Here we have introduced a blood based liquid biopsy that can be utilized for the triage of CT priority when patients present to the ED with an acquired TBI. Alongside the traditional clinical assessment, the ATR-FTIR spectroscopy technique has the potential to aid in clinical decision making, which can save costs and remove radiation risks of unnecessary CT imaging. Stratification of injury patients (both all severities and mild only) versus healthy controls gave excellent sensitivities, specificities, and balanced accuracies. The differentiation between mild patients with one or more CT abnormality versus mild patients with no CT abnormalities provided an accurate identification of 80% of all CT abnormalities. Further, the test is not influenced by patient age or gender.

## Supplementary Material

Supplemental data

Supplemental data

Supplemental data

Supplemental data

Supplemental data

## CENTER-TBI Participants and Investigators

Cecilia Åkerlund,^1^ Krisztina Amrein,^2^ Nada Andelic,^3^ Lasse Andreassen,^4^ Audny Anke,^5^ Anna Antoni,^6^ Gérard Audibert,^7^ Philippe Azouvi,^8^ Maria Luisa Azzolini,^9^ Ronald Bartels,^10^ Pál Barzó,^11^ Romuald Beauvais,^12^ Ronny Beer,^13^ Bo-Michael Bellander,^14^ Antonio Belli,^15^ Habib Benali,^16^ Maurizio Berardino,^17^ Luigi Beretta,^9^ Morten Blaabjerg,^18^ Peter Bragge,^19^ Alexandra Brazinova,^20^ Vibeke Brinck,^21^ Joanne Brooker,^22^ Camilla Brorsson,^23^ Andras Buki,^24^ Monika Bullinger,^25^ Manuel Cabeleira,^26^ Alessio Caccioppola,^27^ Emiliana Calappi,^27^ Maria Rosa Calvi,^9^ Peter Cameron,^28^ Guillermo Carbayo Lozano,^29^ Marco Carbonara,^27^ Simona Cavallo,^17^ Giorgio Chevallard,^30^ Arturo Chieregato,^30^ Giuseppe Citerio,^31,32^ Hans Clusmann,^33^ Mark Coburn,^34^ Jonathan Coles,^35^ Jamie D. Cooper,^36^ Marta Correia,^37^ Amra Čović,^38^ Nicola Curry,^39^ Endre Czeiter^2,4^ Marek Czosnyka,^26^ Claire Dahyot-Fizelier,^40^ Paul Dark,^41^ Helen Dawes,^42^ Véronique De Keyser,^43^ Vincent Degos,^16^ Francesco Della Corte,^44^ Hugo den Boogert,^10^ Bart Depreitere,^45^ Đula Đilvesi,^46^ Abhishek Dixit,^47^ Emma Donoghue,^22^ Jens Dreier,^48^ Guy-Loup Dulière,^49^ Ari Ercole,^47^ Patrick Esser,^42^ Erzsébet Ezer,^50^ Martin Fabricius,^51^ Valery L. Feigin,^52^ Kelly Foks,^53^ Shirin Frisvold,^54^ Alex Furmanov,^55^ Pablo Gagliardo,^56^ Damien Galanaud,^16^ Dashiell Gantner,^28^ Guoyi Gao,^57^ Pradeep George,^58^ Alexandre Ghuysen,^59^ Lelde Giga,^60^ Ben Glocker,^61^ Jagoš Golubovic,^46^ Pedro A. Gomez,^62^ Johannes Gratz,^63^ Benjamin Gravesteijn,^64^ Francesca Grossi,^44^ Russell L. Gruen,^65^ Deepak Gupta,^66^ Juanita A. Haagsma,^64^ Iain Haitsma,^67^ Raimund Helbok,^13^ Eirik Helseth,^68^ Lindsay Horton,^69^ Jilske Huijben,^64^ Peter J. Hutchinson,^70^ Bram Jacobs,^71^ Stefan Jankowski,^72^ Mike Jarrett,^21^ Ji-yao Jiang,^58^ Faye Johnson,^73^ Kelly Jones,^52^ Mladen Karan,^46^ Angelos G. Kolias,^70^ Erwin Kompanje,^74^ Daniel Kondziella,^51^ Evgenios Kornaropoulos,^47^ Lars-Owe Koskinen,^75^ Noémi Kovács,^76^ Ana Kowark,^77^ Alfonso Lagares,^62^ Linda Lanyon,^58^ Steven Laureys,^78^ Fiona Lecky,^79,80^ Didier Ledoux,^78^ Rolf Lefering,^81^ Valerie Legrand,^82^ Aurelie Lejeune,^83^ Leon Levi,^84^ Roger Lightfoot,^85^ Hester Lingsma,^64^ Andrew I.R. Maas,^43^ Ana M. Castaño-León,^62^ Marc Maegele,^86^ Marek Majdan,^20^ Alex Manara,^87^ Geoffrey Manley,^88^ Costanza Martino,^89^ Hugues Maréchal,^49^ Julia Mattern,^90^, Catherine McMahon,^91^ Béla Melegh,^92^ David Menon,^47^ Tomas Menovsky,^43^ Ana Mikolic,^64^ Benoit Misset,^78^ Visakh Muraleedharan,^58^ Lynnette Murray,^28^ Ancuta Negru,^93^ David Nelson,^1^ Virginia Newcombe,^47^ Daan Nieboer,^64^ József Nyirádi,^2^ Otesile Olubukola,^79^ Matej Oresic,^94^ Fabrizio Ortolano,^27^ Aarno Palotie,^95,96,97^ Paul M. Parizel,^98^ Jean-François Payen^99^, Natascha Perera,^12^ Vincent Perlbarg,^16^ Paolo Persona,^100^ Wilco Peul,^101^ Anna Piippo-Karjalainen,^102^ Matti Pirinen,^95^ Dana Pisica,^64^ Horia Ples,^93^ Suzanne Polinder,^64^ Inigo Pomposo,^29^ Jussi P. Posti,^103^ Louis Puybasset,^104^ Andreea Radoi,^105^ Arminas Ragauskas,^106^ Rahul Raj,^102^ Malinka Rambadagalla,^107^ Isabel Retel Helmrich,^64^ Jonathan Rhodes,^108^ Sylvia Richardson,^109^ Sophie Richter,^47^ Samuli Ripatti,^95^ Saulius Rocka,^106^ Cecilie Roe,^110^ Olav Roise,^111,112^ Jonathan Rosand,^113^ Jeffrey V. Rosenfeld,^114^ Christina Rosenlund,^115^ Guy Rosenthal,^55^ Rolf Rossaint,^77^ Sandra Rossi,^100^ Daniel Rueckert,^61^ Martin Rusnák,^116^ Juan Sahuquillo,^105^ Oliver Sakowitz,^90,117^ Renan Sanchez-Porras,^117^ Janos Sandor,^118^ Nadine Schäfer,^81^ Silke Schmidt,^119^ Herbert Schoechl,^120^ Guus Schoonman,^121^ Rico Frederik Schou,^122^ Elisabeth Schwendenwein,^6^ Charlie Sewalt,^64^ Ranjit D. Singh,^101^ Toril Skandsen,^123,124^ Peter Smielewski,^26^ Abayomi Sorinola,^125^ Emmanuel Stamatakis,^47^ Simon Stanworth,^39^ Robert Stevens,^126^ William Stewart,^127^ Ewout W. Steyerberg,^64,128^ Nino Stocchetti,^129^ Nina Sundström,^130^ Riikka Takala,^131^ Viktória Tamás,^125^ Tomas Tamosuitis,^132^ Mark Steven Taylor,^20^ Braden Te Ao,^52^ Olli Tenovuo,^103^ Alice Theadom,^52^ Matt Thomas,^87^ Dick Tibboel,^133^ Marjolein Timmers,^74^ Christos Tolias,^134^ Tony Trapani,^28^ Cristina Maria Tudora,^93^ Andreas Unterberg,^90^ Peter Vajkoczy,^135^ Shirley Vallance,^28^ Egils Valeinis,^60^ Zoltán Vámos,^50^ Mathieu van der Jagt,^136^ Gregory Van der Steen,^43^ Joukje van der Naalt,^71^ Jeroen T.J.M. van Dijck,^101^ Inge A. van Erp,^101^ Thomas A. van Essen,^101^ Wim Van Hecke,^137^ Caroline van Heugten,^138^ Dominique Van Praag,^139^ Ernest van Veen,^64^ Thijs Vande Vyvere,^137^ Roel P. J. van Wijk,^101^ Alessia Vargiolu,^32^ Emmanuel Vega,^83^ Kimberley Velt,^64^ Jan Verheyden,^137^ Paul M. Vespa,^140^ Anne Vik,^123,141^ Rimantas Vilcinis,^132^ Victor Volovici,^67^,Nicole von Steinbüchel,^38^ Daphne Voormolen,^64^ Petar Vulekovic,^46^ Kevin K.W. Wang,^142^ Daniel Whitehouse,^47^ Eveline Wiegers,^64^ Guy Williams,^47^ Lindsay Wilson,^6,9^ Stefan Winzeck,^47^ Stefan Wolf,^143^ Zhihui Yang,^113^ Peter Ylén,^144^ Alexander Younsi,^90^ Frederick A. Zeiler,^47,145^ Veronika Zelinkova,^20^ Agate Ziverte,^60^ Tommaso Zoerle.^27^ The CENTER-TBI Participants and Investigators affiliations may be found online in the Supplementary Material.

## Supplementary Material

Supplementary Figure S3

Supplementary Figure S3CENTER-TBI Participants and Investigators affiliations
