# Supplementary material for: Vibrational Spectroscopy for the Triage of Traumatic Brain Injury Computed Tomography Priority and Hospital Admissions
Source: J Neurotrauma. 2022 Jun 3;39(11-12):773–83. doi: 10.1089/neu.2021.0410 (PMC9225408; doi:10.1089/neu.2021.0410)
Supplement: Supplemental data [file Supp_FigS1.docx]

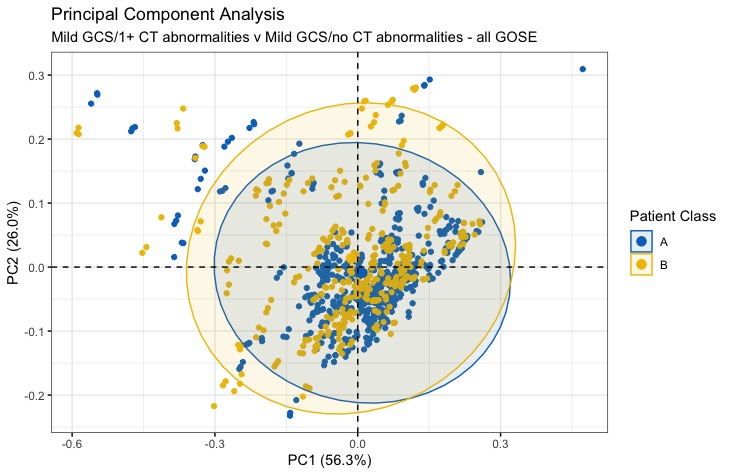


Figure S1: PCA of the first and second dimensions of mild injury patients with at least 1 CT abnormality in blue and mild injury patients with no CT abnormalities in yellow. The eclipses represent a 95% confidence interval. Values in parentheses is the total explained variance in each PC.
