# Supplementary material for: Vibrational Spectroscopy for the Triage of Traumatic Brain Injury Computed Tomography Priority and Hospital Admissions
Source: J Neurotrauma. 2022 Jun 3;39(11-12):773–83. doi: 10.1089/neu.2021.0410 (PMC9225408; doi:10.1089/neu.2021.0410)
Supplement: Supplemental data [file Supp_FigS2.docx]

**
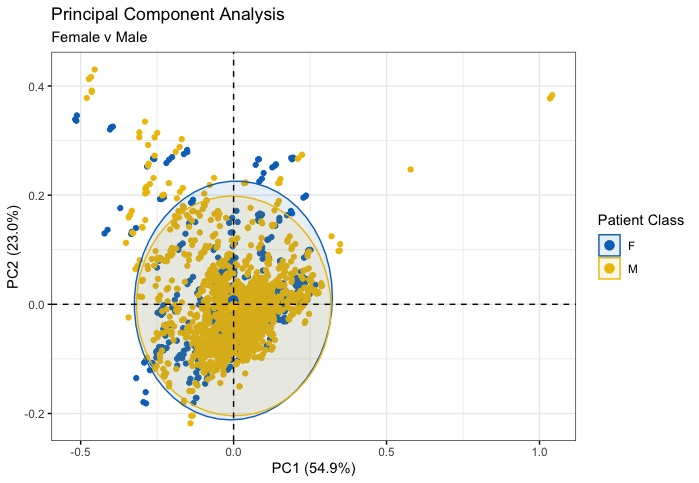
**

Figure S2: PCA of the first and second dimensions with female injury patients in blue and males in yellow. The eclipses represent a 95% confidence interval. Values in parentheses is the total explained variance in each PC.
