# Supplementary material for: Vibrational Spectroscopy for the Triage of Traumatic Brain Injury Computed Tomography Priority and Hospital Admissions
Source: J Neurotrauma. 2022 Jun 3;39(11-12):773–83. doi: 10.1089/neu.2021.0410 (PMC9225408; doi:10.1089/neu.2021.0410)
Supplement: Supplemental data [file Supp_FigS3.docx]

**
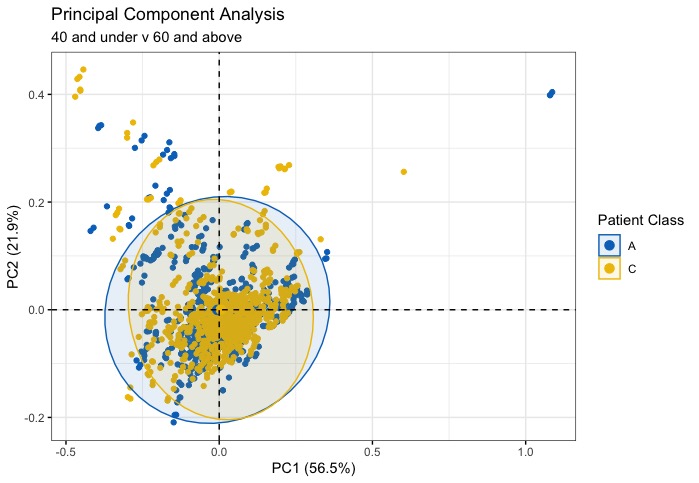
**

Figure S3: PCA of the first and second dimensions with injury patients separated by age. Patients under 40 years old represented in blue and patients 60 and above in yellow. The eclipses represent a 95% confidence interval. Values in parentheses is the total explained variance in each PC.
