# Supplementary material for: Vibrational Spectroscopy for the Triage of Traumatic Brain Injury Computed Tomography Priority and Hospital Admissions
Source: J Neurotrauma. 2022 Jun 3;39(11-12):773–83. doi: 10.1089/neu.2021.0410 (PMC9225408; doi:10.1089/neu.2021.0410)
Supplement: Supplemental data [file Supp_TableS1.docx]

Table S1: Sensitivity, specificity and balanced accuracies for female *versus* male and younger *versus* older head injury patients with the top performing classification model. 95% confidence intervals are included.

|  | **Model** | **Sensitivity (%)** | | | **Specificity (%)** | | | **Balanced accuracy (%)** | | |
| --- | --- | --- | --- | --- | --- | --- | --- | --- | --- | --- |
|  |  | Mean | SD | 95% CI | Mean | SD | 95% CI | Mean | SD | 95% CI |
| Female *vs*. Male | PLS-DA | 50.9 | 10.8 | ±3.0  47.9-53.9 | 62.1 | 7.1 | ±1.9  60.2-64.0 | 56.5 | 6.1 | ±1.7  54.8-58.2 |
| Under 40 *vs.* Over 60 | SVM | 57.0 | 12.6 | ±3.5  53.5-60.5 | 69.6 | 8.9 | ±2.4  67.2-72.0 | 63.3 | 6.0 | ±1.6  61.7-64.9 |
